# [*rac*-1,8-Bis(2-carbamoyleth­yl)-5,5,7,12,12,14-hexa­methyl-1,4,8,11-tetra­aza­cyclo­tetra­deca­ne]copper(II) di­acetate tetra­hydrate: crystal structure and Hirshfeld surface analysis

**DOI:** 10.1107/S2056989021012184

**Published:** 2021-11-23

**Authors:** Sabina Yasmin, Saswata Rabi, Avijit Chakraborty, Huey Chong Kwong, Edward R. T. Tiekink, Tapashi Ghosh Roy

**Affiliations:** a Department of Chemistry, University of Chittagong, Faculty of Science, Chattogarm-4331, Bangladesh; b Department of Chemistry, Chittagong University of Engineering & Technology, Faculty of Engineering & Technology, Chattogarm-4349, Bangladesh; c Research Centre for Crystalline Materials, School of Medical and Life Sciences, Sunway University, 47500 Bandar Sunway, Selangor Darul Ehsan, Malaysia

**Keywords:** crystal structure, copper(II), macrocycle, hydrogen bonding, Hirshfeld surface analysis

## Abstract

The title Cu^II^ macrocyclic complex salt tetra­hydrate, [Cu(C_22_H_46_N_6_O_2_)](C_2_H_3_O_2_)_2_·4H_2_O, sees the metal atom located on a centre of inversion and coordinated within a 4 + 2 (N_4_O_2_) tetra­gonally distorted coordination geometry; the N atoms are derived from the macrocycle and the O atoms from weakly associated [3.2048 (15) Å] acetate anions. Further stability to the three-ion aggregate is provided by intra­molecular amine-N—H⋯O(carboxyl­ate) hydrogen bonds. Hydrogen bonding is also prominent in the mol­ecular packing with amide-N—H⋯O(amide) inter­actions, leading to eight-membered {⋯HNCO}_2_ synthons, amide-N—H⋯O(water), water-O—H⋯O(carboxyl­ate) and water-O—H⋯O(water) hydrogen bonds featuring within the three-dimensional architecture. The calculated Hirshfeld surfaces for the individual components of the asymmetric unit differentiate the water mol­ecules owing to their distinctive supra­molecular association. For each of the anion and cation, H⋯H contacts predominate (50.7 and 65.2%, respectively) followed by H⋯O/O⋯H contacts (44.5 and 29.9%, respectively).

## Abstract 2

The metal ion of the title salt hydrate shows a 4 + 2 (N_4_O_2_) tetra­gonally elongated coordination geometry defined by four macrocyclic-N atoms and two weakly associated acetate-O atoms; the crystal features conventional hydrogen-bonding inter­actions.

## Chemical context

Owing to the multifarious applications of different metal complexes of a wide variety of macrocyclic ligands (Ali *et al.*, 2019 ▸; Bernhardt & Sharpe, 2000 ▸; Lamani *et al.*, 2018 ▸; Vicente *et al.*, 2003 ▸; Xu *et al.*, 2020 ▸), studies on some *N*-pendent macrocyclic ligands and their metal complexes were described by us recently (Dey, Rabi, Hazari *et al.*, 2021 ▸; Dey, Rabi, Palit *et al.*, 2021 ▸). In a continuation of this work, a new *N*-pendent carbamoyl-derived macrocyclic ligand, ‘tet-am’, C_22_H_46_N_6_O_2_, prepared from ‘tet-a′ (an isomeric ligand of the hexa­methyl tetra­zamacrocyclic ligand) and acryl­amide has been synthesized, by employing the procedure described for the preparation of a related *N*-pendent ligand (Dey, Rabi, Hazari *et al.*, 2021 ▸). Thereafter, the inter­action of the new ‘tet-am’ ligand with copper(II) acetate monohydrate furnished violet crystals formulated as [Cu(tet-am)](O_2_CCH_3_)_2_·4H_2_O, hereafter (I). Herein, we describe the synthesis of (I), its analysis by single crystal X-ray diffraction and a detailed study of supra­molecular association by an evaluation of the calculated Hirshfeld surfaces and two-dimensional fingerprint plots. 
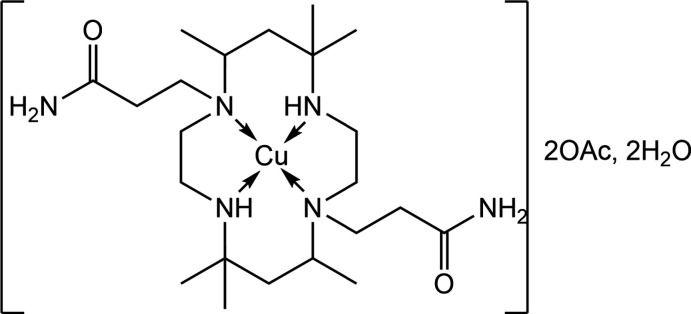


## Structural commentary

**Figure 1:**
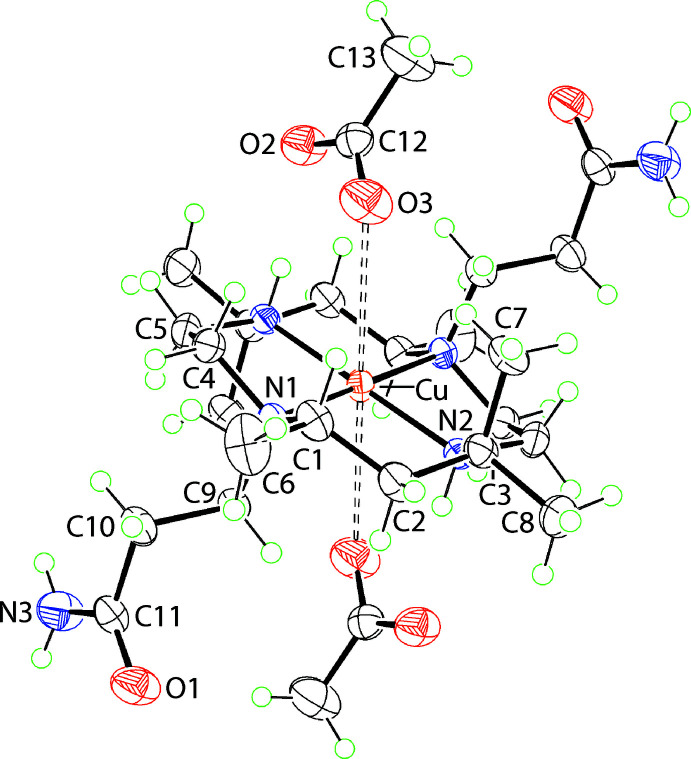
The mol­ecular structure of the complex dication in (I) along with the loosely associated anions, showing the atom-labelling scheme and displacement ellipsoids at the 50% probability level. The mol­ecule is disposed about an inversion centre with unlabelled atoms related by the symmetry operation 1 − *x*, 1 − *y*, 1 − *z*. The weak Cu⋯O3 inter­actions above and below the CuN_4_ plane are shown as dashed lines.

The mol­ecular structure diagram showing the complex dication and loosely associated anions is shown in Fig. 1 ▸. The Cu atom is located on a centre of inversion and is coordinated by tertiary and secondary N atoms with the bond length formed by the former, *i.e*. Cu—N2 = 2.0016 (12) Å, being approximately 0.1 Å shorter than the Cu—N1 bond of 2.1086 (11) Å. Whereas the conformation of the five-membered chelate ring is best described as being an envelope with the C4 atom being the flap atom, the six-membered chelate ring approximates a chair conformation. The acetate anions are weakly associated with the complex cation, forming relatively long Cu⋯O3 separations of 3.2048 (15) Å with extra stability to the three-ion aggregate provided by intra­molecular amine-N—H⋯(carboxyl­ate) hydrogen bonds, Table 1 ▸. The coordination geometry for the Cu centre can therefore, be considered 4 + 2 N_4_O_2_ tetra­gonally distorted. From symmetry, the N1-bound carbamoylethyl groups lie to opposite sides of the CuN_4_ plane and the N1—C9—C10—C11 torsion angle of −178.52 (12)° is consistent with an –anti-periplanar (–ap) configuration.

## Supra­molecular features

**Figure 2:**
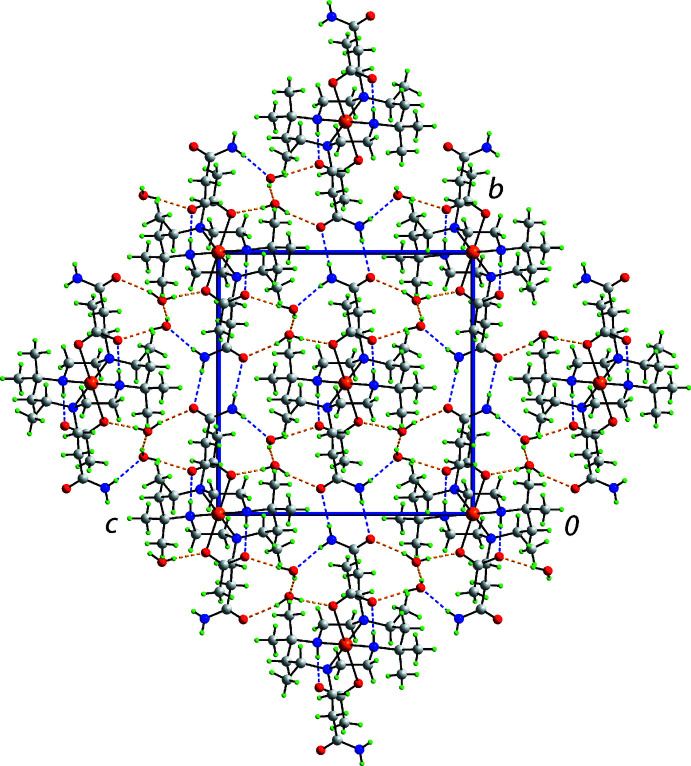
A view of the unit-cell contents of (I) shown in projection down the *a-*axis direction. The O—H⋯O and N—H⋯O hydrogen bonds are shown as orange and blue dashed lines, respectively.

Conventional hydrogen bonding is prominent among the directional supra­molecular contacts evident in the crystal of (I); Table 1 ▸ compiles a listing of these inter­actions. As mentioned above, the amine forms an intra­molecular amine-N—H⋯O(carboxyl­ate) hydrogen bond. The amide forms an amide-N—H⋯O(amide) hydrogen bond with a centrosymmetrically related mol­ecule, *via* an eight-membered {⋯HNCO}_2_ synthon, and the second H atom participates in an amide-N—H⋯O(water) hydrogen bond. This water mol­ecule, *i.e*. water-O1*W*, also forms donor inter­actions to a carboxyl­ate-O2 atom and to the second water mol­ecule, *i.e*. water-O2*W*. The latter forms donor inter­actions with the amide-O and carboxyl­ate-O3 atoms. As can be seen from the unit-cell diagram of Fig. 2 ▸, globally, the three-ion aggregates align in chains along the *a* axis direction with the prominent hydrogen bonds between the mol­ecules in that direction being water-O—H⋯O(water) and water-O—H⋯O3(carboxyl­ate). The remaining hydrogen bonds extend laterally to consolidate the three-dimensional supra­molecular network.

## Analysis of the Hirshfeld surfaces

The Hirshfeld surface analysis for each constituent of (I) was performed to provide further information on the supra­molecular connections in the crystal and to differentiate the modes of association of the water mol­ecules. The calculated Hirshfeld surfaces were mapped over the normalized contact distance *d*_norm_ (Spackman & Jayatilaka, 2009 ▸). These along with the associated two-dimensional fingerprint plots (Spackman & McKinnon, 2002 ▸) were calculated with *Crystal Explorer 17* (Turner *et al.*, 2017 ▸) following literature precedents (Tan *et al.*, 2019 ▸). The colour for the *d*_norm_ surface was scaled between −0.621 (blue) and 1.131 a.u. (red). Key inter­atomic parameters are listed in Table 2 ▸.

**Figure 3:**
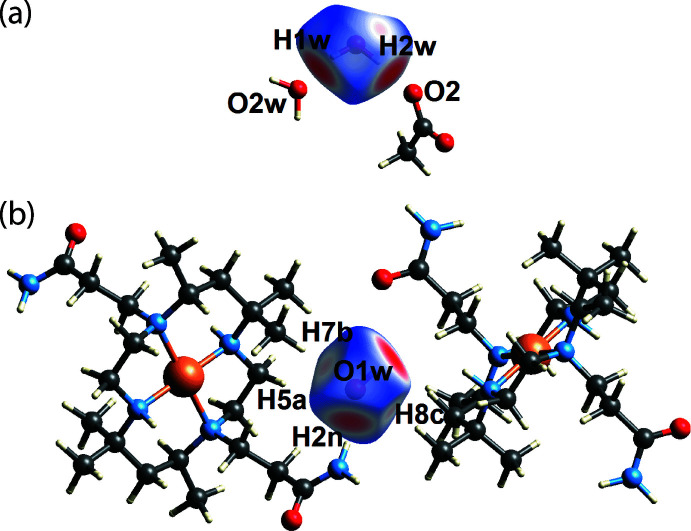
Two views of the Hirshfeld surface for the O1*W*-water mol­ecule of (I) over *d*_norm_ highlighting (*a*) O1*W*—*H*⋯*O*(water/carboxyl­ate) hydrogen bonds and (*b*) amide-N—H⋯O1*W* hydrogen bonds as well as weak C—H⋯O1*W* inter­actions.

**Figure 4:**
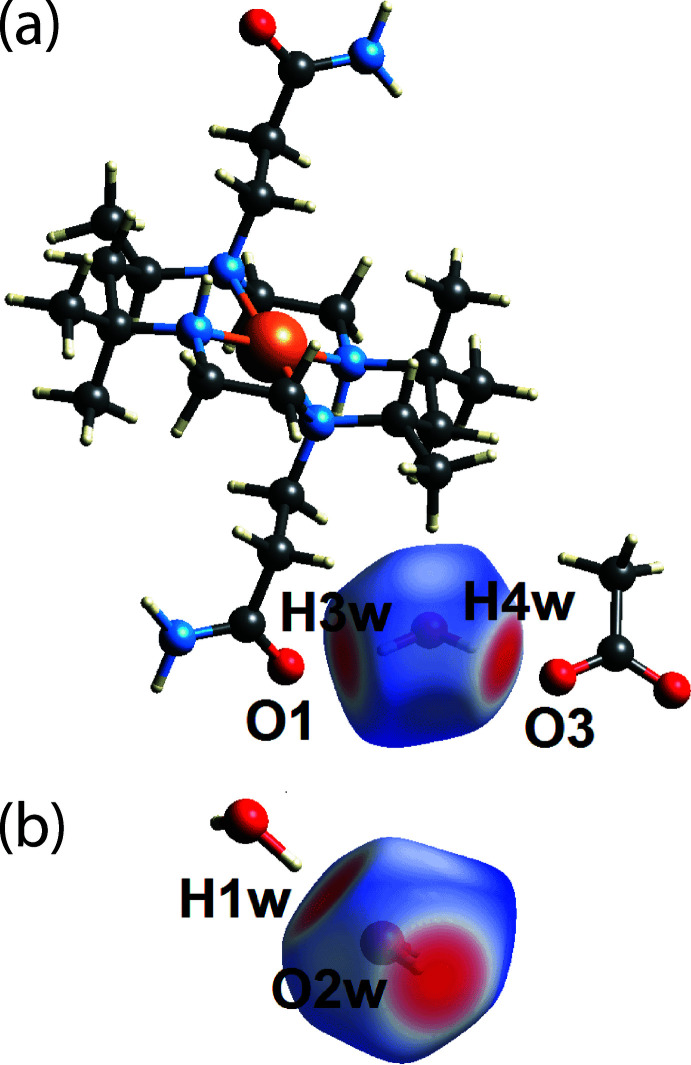
Two views of the Hirshfeld surface for the O2*W*-water mol­ecule of (I) over *d*_norm_ highlighting (*a*) O2*W*—*H*⋯*O*(carbon­yl/carboxyl­ate) hydrogen bonds and (*b*) the O1*W*—H1*W*⋯O2*W* hydrogen bond.

As a hydrogen-bond donor, the two bright red spots on the *d*_norm_-Hirshfeld surface of the O1*W*-water mol­ecule are due to the formation of conventional water-O—H⋯O(water) and water-O—H⋯O(carboxyl­ate) hydrogen bonds, Fig. 3 ▸(*a*). The other bright-red spot appearing on the *d*_norm_-Hirshfeld surface is due to the formation of a conventional primary amide-N—H⋯O(water) hydrogen bond, Fig. 3 ▸(*b*). Further, weak methyl­ene/methyl-C—H⋯O(water) inter­actions are also shown as faint red spots near atoms H5*A*, H7*B* and H8*C* in Fig. 3 ▸(*b*). Similar to the O1*W*-water mol­ecule, the two O2*W*-H atoms participate in conventional water-O—H⋯O(carboxyl­ate) and water-O—H⋯O(amide) hydrogen bonds. These hydrogen bonds are manifested as two bright-red spots on the *d*_norm_-Hirshfeld surface of the O2*W* mol­ecule, Fig. 4 ▸(*a*). The third bright red spot, evident in Fig. 4 ▸(*b*), is due to the water-O—H⋯O(water) hydrogen bond as discussed above.

**Figure 5:**
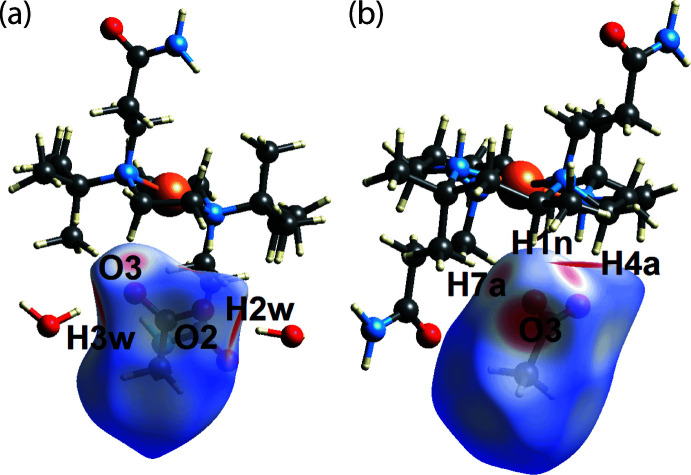
Two views of the Hirshfeld surface for the anion in (I) over *d*_norm_ highlighting (*a*) conventional hydrogen bonds and (*b*) C—H⋯O inter­actions.

For the carboxyl­ate anion, the bright-red spots on its *d*_norm_-Hirshfeld surface correspond to the water-O—H⋯O(carboxyl­ate) hydrogen bonds, Fig. 5 ▸(*a*); the amide-N—H⋯O(carboxyl­ate) hydrogen bond, which also leads to a bright-red spot, is highlighted in Fig. 5 ▸(*b*). At the same time, the weak methyl­ene-H4*A*/methyl-H7*A*⋯O3(carboxyl­ate) inter­actions, with separations of 0.38 and 0.47 Å shorter than the sum of van der Waals radii, respectively, are shown as faint red spots in Fig. 5 ▸(*b*).

**Figure 6:**
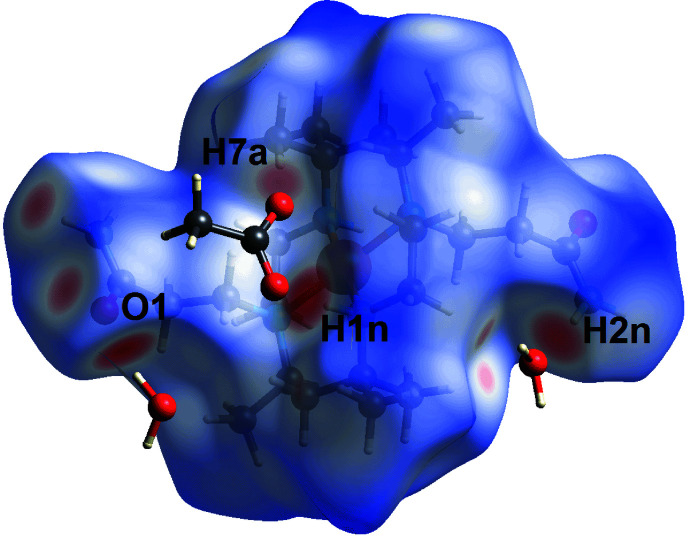
A view of the Hirshfeld surface for the cation in (I) over *d*_norm_.

On the *d*_norm_-Hirshfeld surface calculated for the cation, the bright-red spots near the amide-O1, methyl-H7*A*, amine-H1*N* and amide-H2*N* atoms, Fig. 6 ▸, arise from inter­actions mentioned above.

**Figure 7:**
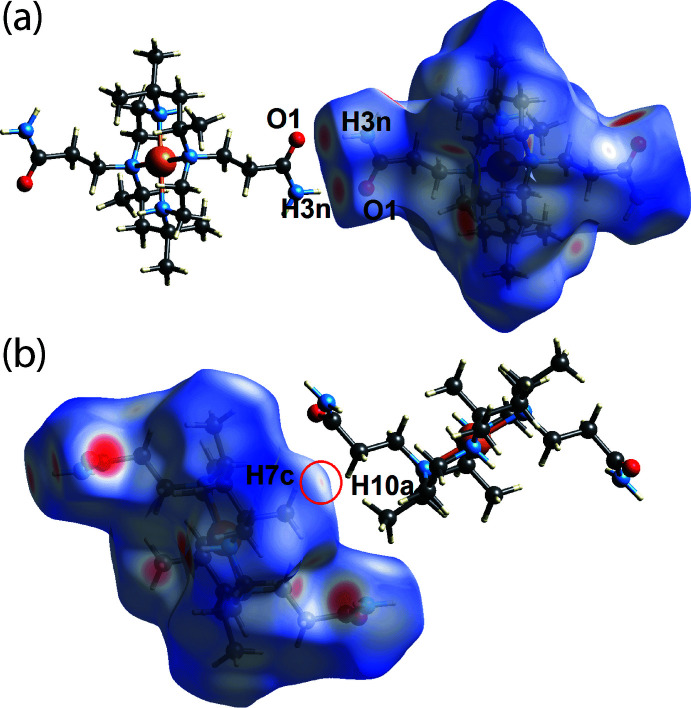
Two views of the Hirshfeld surface for the cation in (I) over *d*_norm_ highlighting (*a*) amide-N—H⋯O(amide) hydrogen bonds and (*b*) H⋯H inter­actions.

The amide-N—H⋯O(amide) hydrogen bond, which serves to link cations, is shown as bright-red spots near the amide-O1 and amide-H3*N* atoms in Fig. 7 ▸(*a*). Especially highlighted in Fig. 7 ▸(*b*) is a short H7*C*⋯H10*A* contact, reflected as a faint-red spot on the *d*_norm_-Hirshfeld surface, with a separation of 2.14 Å, which is 0.26 Å shorter than sum of the van der Waals radii.

**Figure 8:**
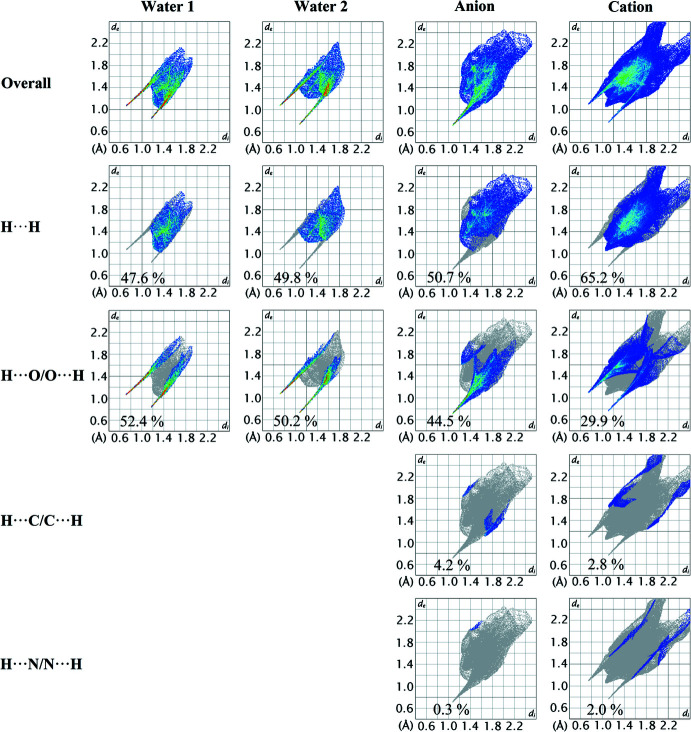
Overall two-dimensional fingerprint plots for each water mol­ecule, anion and cation, and those delineated into H⋯H, H⋯O/O⋯H, H⋯C/C⋯H and H⋯N/N⋯H contacts.

In order to qu­antify the inter­atomic contacts for each individual species comprising the asymmetric-unit, the two-dimensional fingerprint plots were also generated. The overall fingerprint plot and those delineated into H⋯H, H⋯O/O⋯H, H⋯C/C⋯H and H⋯N/N⋯H surface contacts are illustrated in Fig. 8 ▸, and the percentage contributions of the delineated contacts are tabulated in Table 3 ▸. As each water mol­ecule only inter­acts with hydrogen and oxygen atoms, their two-dimensional fingerprint plot are saturated by H⋯H and H⋯O/O⋯H contacts. For the O1*W*-mol­ecule, the H⋯H and H⋯O/O⋯H contacts contribute 47.6 and 52.4% to the Hirshfeld surface, respectively. On account of the C—H⋯O and N—H⋯O inter­actions evinced for the O1*W*-mol­ecule, the contribution of H⋯H and H⋯O/O⋯H contacts differ by ∼2% as compared to the near equal contributions for the O2*W*-mol­ecule (H⋯H = 49.8%: H⋯O/O⋯H = 50.2%), which does not form analogous contacts.

The most significant inter­molecular contacts involving the anion are the H⋯H and H⋯O/O⋯H contacts; these contacts tipped at *d*_e_ = *d*_i_ ∼ 2.2 Å and *d*_e_ + *d*_i_ ∼ 1.8 Å, respectively, Fig. 8 ▸. The H⋯H contacts contribute 50.7% to the overall Hirshfeld surface of the anion, while the H⋯O/O⋯H contribute 44.5%, Table 3 ▸. The next most significant inter­atomic contacts are H⋯C/C⋯H contacts, but these only contribute 4.2% to the overall Hirshfeld surface. Consistent with the loose association between the anion and cation, the O⋯Cu/Cu⋯O contacts only contribute 0.3% to the overall Hirshfeld surface, Table 3 ▸.

For the cation, H⋯H contacts contribute 65.2% to the overall Hirshfeld surface with the shortest contact, manifested in the beak-like peak tipped at *d*_e_ = *d*_i_ ∼2.2 Å, Fig. 8 ▸, corres­ponding to the H7*C*⋯H10*A* contact listed in Table 2 ▸. The H⋯O/O⋯H contacts contribute 29.9% to the surface reflecting the conventional hydrogen bonds that involve water, acetate and carbamoylethyl moieties, as discussed above. The shortest H⋯O/O⋯H contacts are reflected as two sharp spikes at *d*_e_ = *d*_i_ ∼1.9 Å in Fig. 8 ▸. Even through both H⋯C/C⋯H and H⋯N/N⋯H contacts appear in the two-dimensional fingerprint plots of the cation, their contributions to the overall Hirshfeld surface are only 2.8 and 2.0%, respectively. As observed for the anion, the weak connection between the Cu^II^ centre and the carboxyl­ate ligand is reflected in a very low contribution of O⋯Cu/Cu⋯O contacts (0.1%) to the overall Hirshfeld surface of the cation.

## Database survey

There are two relevant structures in the literature available for comparison having closely related 14-membered tetra­aza macrocycles bearing two pendent N-bound CH_2_CH_2_CONH_2_ arms (Kang *et al.*, 2008 ▸). These structures present very different coordination geometries to each other and to that of (I). The common feature of the literature structures is the presence of perchlorate counter-anions, which do not coordinate the Cu^II^ atom in either case. Rather, the amide-O atom of one side-arm folds over the mol­ecule to form a Cu—O bond. In the C-*rac*-macrocyclic complex, a square-pyramidal geometry ensues with the amide-O atom [2.207 (4) Å] occupying the apical position. While the *trans*-orientated Cu—N(tertiary) bond lengths of 2.083 (4) and 2.086 (4) Å are longer than Cu—N(secondary) bonds of 2.035 (4) and 2.045 (4) Å, the differences between the short and long bond lengths are not as great as noted above for (I). In the structure with the configurational C-*meso* isomer, the coordination geometry changes to trigonal-bipyramidal with the amide-O atom occupying an equatorial position, forming a significantly shorter Cu—O bond length [2.007 (4) Å] compared to that in the racemic isomer. The tertiary-N atoms occupy axial positions and form Cu—N(tertiary) bond lengths of 2.063 (4) and 2.088 (4) Å which overlap with the Cu—N(secondary) bond lengths of 2.077 (4) and 2.090 (3) Å. The foregoing demonstrates a dependency of the Cu atom coordination geometry and the magnitudes of putative Cu to O inter­actions on the nature of the counter-anion and isomeric form of the ligand.

## Synthesis and crystallization

**Synthesis of** ***N*** **-carbamoylethyl pendent derivative (tet-am):** The isomeric ligand, tet-a (0.320 g, 1.0 mmol), dissolved in hot methanol (50 ml), and acryl­amide (0.28 g, 4.0 mmol), taken in a minimum amount of hot methanol, were mixed. The reaction mixture was refluxed for about 12 h, cooled to room temperature, filtered and allowed to stand for three days to evaporate slowly. The white product that formed, tet-am, was separated by filtration, washed with methanol followed by water and finally dried in a desiccator over silica gel; m.p. 458 K.

**[Cu(tet-am)](O_2_CCH_3_)_2_·4H_2_O (I):** The macrocycle, tet-am (0.426 g, 1.0 mmol) and copper(II) acetate monohydrate (0.199 g, 1.0 mmol) were dissolved separately in hot methanol (25 ml) and mixed while hot, resulting in an immediate colour change. The solution was heated on a steam-bath until the volume was reduced to less than 10 ml. After standing overnight, the sticky material that had formed was dissolved in a minimum amount of ethanol followed by the addition of excess di­ethyl­ether. The liquid portion was deca­nted and the remaining violet precipitate, (I), was dried over silica gel and stored in a vacuum desiccator. Some violet crystals suitable for X-ray analysis were collected from the mother liquor (ethanol + di­ethyl­ether) during the isolation of the complex; m.p. 378 K.

## Refinement

Crystal data, data collection and structure refinement details are summarized in Table 4 ▸. The carbon-bound H atoms were placed in calculated positions (C—H = 0.96–0.98 Å) and were included in the refinement in the riding-model approximation, with *U*_iso_(H) set to 1.2–1.5*U*_eq_(C). The O- and N-bound H atoms were located in a difference-Fourier map and were refined with O—H = 0.82±0.01 and N—H = 0.86±0.01 Å distance restraints, and with *U*_iso_(H) set to 1.5*U*_eq_(O) and 1.2*U*_eq_(N), respectively.

## Supplementary Material

Crystal structure: contains datablock(s) I, global. DOI: 10.1107/S2056989021012184/hb8001sup1.cif

Structure factors: contains datablock(s) I. DOI: 10.1107/S2056989021012184/hb8001Isup2.hkl

CCDC reference: 2122547

Additional supporting information:  crystallographic information; 3D view; checkCIF report

## Figures and Tables

**Table 1:** Hydrogen-bond geometry (Å, °)

*D*—H⋯*A*	*D*—H	H⋯*A*	*D*⋯*A*	*D*—H⋯*A*
N2—H1*N*⋯O2^i^	0.87 (1)	2.00 (1)	2.8634 (18)	173 (2)
N3—H2*N*⋯O1*W* ^ii^	0.85 (2)	2.19 (2)	3.031 (2)	170 (2)
N3—H3*N*⋯O1^iii^	0.86 (2)	2.37 (2)	3.221 (2)	175 (1)
O1*W*—H2*W*⋯O2	0.83 (1)	1.94 (1)	2.7574 (18)	167 (2)
O1*W*—H1*W*⋯O2*W* ^iv^	0.83 (2)	1.98 (2)	2.794 (2)	167 (2)
O2*W*—H3*W*⋯O3	0.83 (2)	1.96 (2)	2.779 (2)	169 (2)
O2*W*—H4*W*⋯O1^v^	0.82 (2)	2.06 (2)	2.869 (2)	168 (2)

Symmetry codes: (i) -x+1, -y+1, -z+1; (ii) -x+{\script{1\over 2}}, y+{\script{1\over 2}}, -z+{\script{3\over 2}}; (iii) -x+1, -y+2, -z+1; (iv) x+{\script{1\over 2}}, -y+{\script{1\over 2}}, z+{\script{1\over 2}}; (v) -x+{\script{1\over 2}}, y-{\script{1\over 2}}, -z+{\script{1\over 2}}.

**Table 2:** A summary of short inter­atomic contacts (Å) for (I)^*a*^

Contact	Distance	Symmetry operation
O1*W*—H1*W*⋯O2*W* ^*b*^	1.83	*x* + {1\over 2}, −*y* + {1\over 2}, *z* + {1\over 2}
O1*W*—H2*W*⋯O2^*b*^	1.79	*x*, *y*, *z*
N3—H2*N*⋯O1*W* ^*b*^	2.03	−*x* + {1\over 2}, *y* + {1\over 2}, −*z* + {3\over 2}
O2*W*—H3*W*⋯O3^*b*^	1.81	*x*, *y*, *z*
O2*W*—H4*W*⋯O1^*b*^	1.90	−*x* + {1\over 2}, *y* − {1\over 2}, −*z* + {1\over 2}
N2—H1*N*⋯O2^*b*^	1.86	−*x* + 1, −*y* + 1, −*z* + 1
N3—H3*N*⋯O1^*b*^	2.22	−*x* + 1, −*y* + 2, −*z* + 1
C5—H5*A*⋯O1*W*	2.38	−*x* + {1\over 2}, *y* + {1\over 2}, −*z* + {3\over 2}
C7—H7*B*⋯O1*W*	2.45	*x* + {1\over 2}, −*y* + {1\over 2}, *z* − {1\over 2}
C8—H8*C*⋯O1*W*	2.48	−*x* + 1, −*y* + 1, −*z* + 1
C4—H4*A*⋯O3	2.35	*x*, *y*, *z*
C7—H7*A*⋯O3	2.26	*x*, *y*, *z*
H7*C*⋯H10*A*	2.14	−*x* + {1\over 2}, *y* − {1\over 2}, −*z* + {1\over 2}

Notes: (*a*) The inter­atomic distances are calculated in *Crystal Explorer 17* (Turner *et al.*, 2017 ▸) with the *X*—H bond lengths adjusted to their neutron values. (*b*) The inter­action corresponds to a conventional hydrogen bond (compare Table 1 ▸).

**Table 3:** A summary of the percentage contributions to the calculated Hirshfeld surfaces for the individual constituents of (I)

Contact	Water-O1*W*	Water-O2*W*	Anion	Cation
H⋯H	47.6	49.8	50.7	65.2
H⋯O/O⋯H	52.4	50.2	44.5	29.9
H⋯C/C⋯H	–	–	4.2	2.8
H⋯N/N⋯H	–	–	0.3	2.0
C⋯Cu/Cu⋯C	–	–	0.3	0.1

**Table 4:** Experimental details

Crystal data	
Chemical formula	[Cu(C_22_H_46_N_6_O_2_)](C_2_H_3_O_2_)_2_·4H_2_O
*M*_r_	680.34
Crystal system, space group	Monoclinic, *P*2_1_/*n*
Temperature (K)	298
*a*, *b*, *c* (Å)	8.5733 (1), 14.2616 (2), 13.9853 (1)
β (°)	97.525 (1)
*V* (Å^3^)	1695.24 (3)
*Z*	2
Radiation type	Cu *K*α
μ (mm^−1^)	1.41
Crystal size (mm)	0.13 × 0.10 × 0.09

Data collection	
Diffractometer	XtaLAB Synergy, Dualflex, AtlasS2
Absorption correction	Gaussian (*CrysAlis PRO*; Rigaku OD, 2018 ▸)
*T*_min_, *T*_max_	0.759, 1.000
No. of measured, independent and observed [*I* > 2σ(*I*)] reflections	21762, 3022, 2902
*R*_int_	0.021
(sin θ/λ)_max_ (Å^−1^)	0.597

Refinement	
*R*[*F* ^2^ > 2σ(*F* ^2^)], *wR*(*F* ^2^), *S*	0.029, 0.082, 1.05
No. of reflections	3022
No. of parameters	221
No. of restraints	9
H-atom treatment	H atoms treated by a mixture of independent and constrained refinement
Δρ_max_, Δρ_min_ (e Å^−3^)	0.27, −0.37

Computer programs: *CrysAlis PRO* (Rigaku OD, 2018 ▸), *SHELXS* (Sheldrick, 2015*a* ▸), *SHELXL2018/3* (Sheldrick, 2015*b* ▸), *ORTEP-3 for Windows* (Farrugia, 2012 ▸), *DIAMOND* (Brandenburg, 2006 ▸) and *publCIF* (Westrip, 2010 ▸).

## supplementary crystallographic information

### Crystal data

**Table:**

[Cu(C_22_H_46_N_6_O_2_)](C_2_H_3_O_2_)_2_·4H_2_O	*F*(000) = 734
*M**_r_* = 680.34	*D*_x_ = 1.333 Mg m^−^^3^
Monoclinic, *P*2_1_/*n*	Cu *K*α radiation, λ = 1.54184 Å
*a* = 8.5733 (1) Å	Cell parameters from 15564 reflections
*b* = 14.2616 (2) Å	θ = 4.5–76.7°
*c* = 13.9853 (1) Å	µ = 1.41 mm^−^^1^
β = 97.525 (1)°	*T* = 298 K
*V* = 1695.24 (3) Å^3^	Prism, violet
*Z* = 2	0.13 × 0.10 × 0.09 mm

### Data collection

**Table:**

XtaLAB Synergy, Dualflex, AtlasS2 diffractometer	2902 reflections with *I* > 2σ(*I*)
Detector resolution: 5.2558 pixels mm^-1^	*R*_int_ = 0.021
ω scans	θ_max_ = 67.1°, θ_min_ = 4.5°
Absorption correction: gaussian (*CrysAlis PRO*; Rigaku OD, 2018)	*h* = −10→10
*T*_min_ = 0.759, *T*_max_ = 1.000	*k* = −17→15
21762 measured reflections	*l* = −16→16
3022 independent reflections	

### Refinement

**Table:**

Refinement on *F*^2^	Primary atom site location: structure-invariant direct methods
Least-squares matrix: full	Secondary atom site location: difference Fourier map
*R*[*F*^2^ > 2σ(*F*^2^)] = 0.029	Hydrogen site location: mixed
*wR*(*F*^2^) = 0.082	H atoms treated by a mixture of independent and constrained refinement
*S* = 1.05	*w* = 1/[σ^2^(*F*_o_^2^) + (0.044*P*)^2^ + 0.7827*P*] where *P* = (*F*_o_^2^ + 2*F*_c_^2^)/3
3022 reflections	(Δ/σ)_max_ < 0.001
221 parameters	Δρ_max_ = 0.27 e Å^−^^3^
9 restraints	Δρ_min_ = −0.37 e Å^−^^3^

### Special details

**Table:**

Geometry. All esds (except the esd in the dihedral angle between two l.s. planes) are estimated using the full covariance matrix. The cell esds are taken into account individually in the estimation of esds in distances, angles and torsion angles; correlations between esds in cell parameters are only used when they are defined by crystal symmetry. An approximate (isotropic) treatment of cell esds is used for estimating esds involving l.s. planes.

### Fractional atomic coordinates and isotropic or equivalent isotropic displacement parameters (Å^2^)

**Table:**

	*x*	*y*	*z*	*U*_iso_*/*U*_eq_	
Cu	0.500000	0.500000	0.500000	0.02360 (11)	
O1	0.43622 (17)	0.90346 (9)	0.40245 (9)	0.0489 (3)	
N1	0.33373 (13)	0.59384 (8)	0.42865 (8)	0.0240 (3)	
N2	0.62472 (15)	0.49154 (8)	0.38892 (9)	0.0247 (3)	
H1N	0.6690 (19)	0.5461 (8)	0.3906 (12)	0.030*	
N3	0.3815 (2)	0.90050 (11)	0.55447 (11)	0.0467 (4)	
H2N	0.336 (2)	0.8725 (14)	0.5972 (13)	0.056*	
H3N	0.424 (2)	0.9545 (10)	0.5645 (16)	0.056*	
C1	0.28843 (18)	0.56941 (11)	0.32369 (11)	0.0306 (3)	
H1	0.242073	0.506533	0.322290	0.037*	
C2	0.4283 (2)	0.56331 (11)	0.26753 (10)	0.0321 (3)	
H2A	0.489347	0.620338	0.279548	0.039*	
H2B	0.388224	0.562360	0.199376	0.039*	
C3	0.54004 (19)	0.48013 (10)	0.28817 (10)	0.0280 (3)	
C4	0.19566 (17)	0.57681 (11)	0.48095 (11)	0.0300 (3)	
H4A	0.148419	0.516840	0.461466	0.036*	
H4B	0.117429	0.625285	0.464338	0.036*	
C5	0.24546 (18)	0.57697 (11)	0.58791 (11)	0.0316 (3)	
H5A	0.280725	0.639145	0.608960	0.038*	
H5B	0.157198	0.559990	0.621238	0.038*	
C6	0.1631 (2)	0.63392 (15)	0.26975 (13)	0.0509 (5)	
H6A	0.079754	0.643442	0.308328	0.076*	
H6B	0.121476	0.605451	0.209592	0.076*	
H6C	0.209854	0.693189	0.257616	0.076*	
C7	0.4507 (2)	0.38741 (11)	0.27926 (12)	0.0375 (4)	
H7A	0.391849	0.380632	0.332703	0.056*	
H7B	0.524106	0.336540	0.279424	0.056*	
H7C	0.380039	0.386682	0.220060	0.056*	
C8	0.6593 (2)	0.48262 (13)	0.21532 (13)	0.0421 (4)	
H8A	0.604261	0.482721	0.150975	0.063*	
H8B	0.726044	0.428407	0.224151	0.063*	
H8C	0.722305	0.538298	0.225308	0.063*	
C9	0.40326 (17)	0.68993 (10)	0.44700 (10)	0.0265 (3)	
H9A	0.468591	0.703235	0.397011	0.032*	
H9B	0.471905	0.688257	0.507891	0.032*	
C10	0.28820 (19)	0.77189 (11)	0.45057 (12)	0.0351 (4)	
H10A	0.217963	0.774789	0.390345	0.042*	
H10B	0.224957	0.761536	0.502257	0.042*	
C11	0.37579 (19)	0.86430 (11)	0.46701 (11)	0.0325 (3)	
O2	0.20199 (16)	0.33641 (9)	0.60394 (9)	0.0456 (3)	
O3	0.21974 (19)	0.35212 (10)	0.44993 (9)	0.0552 (4)	
C12	0.19402 (18)	0.30430 (12)	0.52047 (11)	0.0332 (3)	
C13	0.1550 (3)	0.20209 (15)	0.50445 (17)	0.0624 (6)	
H13A	0.096896	0.193856	0.441527	0.094*	
H13B	0.092734	0.180918	0.552479	0.094*	
H13C	0.250589	0.166327	0.509114	0.094*	
O1W	0.25081 (16)	0.28246 (9)	0.79485 (9)	0.0454 (3)	
H1W	0.3363 (17)	0.2549 (16)	0.7996 (15)	0.068*	
H2W	0.222 (3)	0.2958 (17)	0.7377 (9)	0.068*	
O2W	0.02782 (18)	0.31581 (13)	0.27799 (11)	0.0638 (4)	
H3W	0.094 (3)	0.329 (2)	0.3246 (12)	0.096*	
H4W	0.039 (3)	0.3485 (19)	0.2309 (13)	0.096*	

### Atomic displacement parameters (Å^2^)

**Table:**

	*U*^11^	*U*^22^	*U*^33^	*U*^12^	*U*^13^	*U*^23^
Cu	0.02688 (18)	0.02365 (18)	0.02075 (17)	0.00575 (11)	0.00493 (12)	0.00138 (10)
O1	0.0774 (9)	0.0367 (7)	0.0346 (6)	−0.0117 (6)	0.0152 (6)	−0.0025 (5)
N1	0.0245 (6)	0.0231 (6)	0.0242 (6)	0.0023 (5)	0.0019 (5)	−0.0001 (5)
N2	0.0294 (7)	0.0208 (6)	0.0246 (6)	0.0000 (5)	0.0061 (5)	−0.0008 (5)
N3	0.0684 (11)	0.0400 (9)	0.0332 (8)	−0.0110 (8)	0.0125 (7)	−0.0059 (6)
C1	0.0347 (8)	0.0287 (8)	0.0262 (7)	0.0022 (6)	−0.0044 (6)	−0.0041 (6)
C2	0.0471 (9)	0.0279 (8)	0.0210 (7)	0.0026 (7)	0.0028 (6)	0.0010 (6)
C3	0.0393 (8)	0.0244 (7)	0.0210 (7)	−0.0010 (6)	0.0064 (6)	−0.0023 (6)
C4	0.0240 (7)	0.0282 (8)	0.0381 (8)	0.0019 (6)	0.0048 (6)	0.0019 (6)
C5	0.0309 (8)	0.0312 (8)	0.0349 (8)	0.0060 (6)	0.0124 (6)	0.0013 (6)
C6	0.0510 (11)	0.0608 (12)	0.0353 (9)	0.0186 (9)	−0.0148 (8)	−0.0050 (8)
C7	0.0434 (9)	0.0276 (8)	0.0404 (9)	−0.0035 (7)	0.0013 (7)	−0.0061 (7)
C8	0.0596 (11)	0.0403 (9)	0.0299 (8)	0.0007 (8)	0.0195 (8)	−0.0022 (7)
C9	0.0282 (7)	0.0235 (7)	0.0273 (7)	0.0010 (6)	0.0016 (5)	−0.0012 (6)
C10	0.0358 (8)	0.0258 (8)	0.0423 (9)	0.0045 (6)	0.0000 (7)	−0.0005 (7)
C11	0.0404 (8)	0.0246 (8)	0.0320 (8)	0.0054 (6)	0.0023 (6)	0.0006 (6)
O2	0.0578 (8)	0.0461 (7)	0.0336 (6)	−0.0087 (6)	0.0081 (5)	0.0003 (5)
O3	0.0862 (10)	0.0462 (8)	0.0341 (7)	−0.0067 (7)	0.0110 (6)	0.0054 (6)
C12	0.0292 (8)	0.0353 (8)	0.0347 (8)	−0.0005 (6)	0.0024 (6)	0.0019 (7)
C13	0.0832 (16)	0.0387 (11)	0.0644 (13)	−0.0094 (10)	0.0066 (12)	−0.0019 (10)
O1W	0.0582 (8)	0.0419 (7)	0.0389 (7)	0.0020 (6)	0.0169 (6)	0.0080 (5)
O2W	0.0590 (9)	0.0902 (12)	0.0417 (8)	−0.0211 (8)	0.0049 (6)	0.0076 (8)

### Geometric parameters (Å, º)

**Table:**

Cu—N2	2.0016 (12)	C5—H5B	0.9700
Cu—N2^i^	2.0016 (12)	C6—H6A	0.9600
Cu—N1	2.1086 (11)	C6—H6B	0.9600
Cu—N1^i^	2.1086 (11)	C6—H6C	0.9600
O1—C11	1.232 (2)	C7—H7A	0.9600
N1—C4	1.4906 (18)	C7—H7B	0.9600
N1—C9	1.5031 (18)	C7—H7C	0.9600
N1—C1	1.5092 (18)	C8—H8A	0.9600
N2—C5^i^	1.4846 (19)	C8—H8B	0.9600
N2—C3	1.5070 (19)	C8—H8C	0.9600
N2—H1N	0.864 (9)	C9—C10	1.535 (2)
N3—C11	1.323 (2)	C9—H9A	0.9700
N3—H2N	0.854 (10)	C9—H9B	0.9700
N3—H3N	0.857 (10)	C10—C11	1.519 (2)
C1—C2	1.519 (2)	C10—H10A	0.9700
C1—C6	1.535 (2)	C10—H10B	0.9700
C1—H1	0.9800	O2—C12	1.247 (2)
C2—C3	1.529 (2)	O3—C12	1.243 (2)
C2—H2A	0.9700	C12—C13	1.506 (3)
C2—H2B	0.9700	C13—H13A	0.9600
C3—C7	1.525 (2)	C13—H13B	0.9600
C3—C8	1.536 (2)	C13—H13C	0.9600
C4—C5	1.501 (2)	O1W—H1W	0.827 (9)
C4—H4A	0.9700	O1W—H2W	0.828 (9)
C4—H4B	0.9700	O2W—H3W	0.827 (10)
C5—H5A	0.9700	O2W—H4W	0.823 (10)
			
N2—Cu—N2^i^	180.0	N2^i^—C5—H5B	109.9
N2—Cu—N1	93.88 (5)	C4—C5—H5B	109.9
N2^i^—Cu—N1	86.12 (5)	H5A—C5—H5B	108.3
N2—Cu—N1^i^	86.12 (5)	C1—C6—H6A	109.5
N2^i^—Cu—N1^i^	93.88 (5)	C1—C6—H6B	109.5
N1—Cu—N1^i^	180.00 (5)	H6A—C6—H6B	109.5
C4—N1—C9	112.80 (11)	C1—C6—H6C	109.5
C4—N1—C1	108.65 (11)	H6A—C6—H6C	109.5
C9—N1—C1	114.98 (11)	H6B—C6—H6C	109.5
C4—N1—Cu	101.27 (8)	C3—C7—H7A	109.5
C9—N1—Cu	105.63 (8)	C3—C7—H7B	109.5
C1—N1—Cu	112.69 (8)	H7A—C7—H7B	109.5
C5^i^—N2—C3	112.86 (11)	C3—C7—H7C	109.5
C5^i^—N2—Cu	109.37 (9)	H7A—C7—H7C	109.5
C3—N2—Cu	119.42 (9)	H7B—C7—H7C	109.5
C5^i^—N2—H1N	105.8 (12)	C3—C8—H8A	109.5
C3—N2—H1N	106.2 (11)	C3—C8—H8B	109.5
Cu—N2—H1N	101.6 (11)	H8A—C8—H8B	109.5
C11—N3—H2N	119.9 (16)	C3—C8—H8C	109.5
C11—N3—H3N	117.7 (15)	H8A—C8—H8C	109.5
H2N—N3—H3N	122 (2)	H8B—C8—H8C	109.5
N1—C1—C2	113.33 (12)	N1—C9—C10	117.24 (12)
N1—C1—C6	114.45 (13)	N1—C9—H9A	108.0
C2—C1—C6	109.21 (14)	C10—C9—H9A	108.0
N1—C1—H1	106.4	N1—C9—H9B	108.0
C2—C1—H1	106.4	C10—C9—H9B	108.0
C6—C1—H1	106.4	H9A—C9—H9B	107.2
C1—C2—C3	117.68 (13)	C11—C10—C9	111.01 (13)
C1—C2—H2A	107.9	C11—C10—H10A	109.4
C3—C2—H2A	107.9	C9—C10—H10A	109.4
C1—C2—H2B	107.9	C11—C10—H10B	109.4
C3—C2—H2B	107.9	C9—C10—H10B	109.4
H2A—C2—H2B	107.2	H10A—C10—H10B	108.0
N2—C3—C7	110.20 (12)	O1—C11—N3	122.17 (16)
N2—C3—C2	107.82 (11)	O1—C11—C10	121.96 (14)
C7—C3—C2	111.22 (13)	N3—C11—C10	115.87 (15)
N2—C3—C8	109.67 (13)	O3—C12—O2	123.22 (16)
C7—C3—C8	109.68 (13)	O3—C12—C13	118.17 (16)
C2—C3—C8	108.20 (13)	O2—C12—C13	118.59 (16)
N1—C4—C5	110.38 (12)	C12—C13—H13A	109.5
N1—C4—H4A	109.6	C12—C13—H13B	109.5
C5—C4—H4A	109.6	H13A—C13—H13B	109.5
N1—C4—H4B	109.6	C12—C13—H13C	109.5
C5—C4—H4B	109.6	H13A—C13—H13C	109.5
H4A—C4—H4B	108.1	H13B—C13—H13C	109.5
N2^i^—C5—C4	108.89 (12)	H1W—O1W—H2W	109.8 (15)
N2^i^—C5—H5A	109.9	H3W—O2W—H4W	111.1 (16)
C4—C5—H5A	109.9		
			
C4—N1—C1—C2	−167.31 (12)	C1—C2—C3—N2	−68.02 (17)
C9—N1—C1—C2	65.21 (15)	C1—C2—C3—C7	52.90 (18)
Cu—N1—C1—C2	−55.92 (14)	C1—C2—C3—C8	173.43 (13)
C4—N1—C1—C6	66.55 (17)	C9—N1—C4—C5	−65.30 (15)
C9—N1—C1—C6	−60.92 (18)	C1—N1—C4—C5	165.99 (12)
Cu—N1—C1—C6	177.94 (12)	Cu—N1—C4—C5	47.15 (12)
N1—C1—C2—C3	71.09 (17)	N1—C4—C5—N2^i^	−54.16 (16)
C6—C1—C2—C3	−160.03 (14)	C4—N1—C9—C10	−40.36 (17)
C5^i^—N2—C3—C7	67.35 (16)	C1—N1—C9—C10	84.99 (15)
Cu—N2—C3—C7	−63.28 (14)	Cu—N1—C9—C10	−150.11 (11)
C5^i^—N2—C3—C2	−171.10 (12)	N1—C9—C10—C11	−178.52 (12)
Cu—N2—C3—C2	58.28 (14)	C9—C10—C11—O1	75.2 (2)
C5^i^—N2—C3—C8	−53.49 (16)	C9—C10—C11—N3	−105.52 (17)
Cu—N2—C3—C8	175.88 (10)		

Symmetry code: (i) −*x*+1, −*y*+1, −*z*+1.

### Hydrogen-bond geometry (Å, º)

**Table:**

*D*—H···*A*	*D*—H	H···*A*	*D*···*A*	*D*—H···*A*
N2—H1*N*···O2^i^	0.87 (1)	2.00 (1)	2.8634 (18)	173 (2)
N3—H2*N*···O1*W*^ii^	0.85 (2)	2.19 (2)	3.031 (2)	170 (2)
N3—H3*N*···O1^iii^	0.86 (2)	2.37 (2)	3.221 (2)	175 (1)
O1*W*—H2*W*···O2	0.83 (1)	1.94 (1)	2.7574 (18)	167 (2)
O1*W*—H1*W*···O2*W*^iv^	0.83 (2)	1.98 (2)	2.794 (2)	167 (2)
O2*W*—H3*W*···O3	0.83 (2)	1.96 (2)	2.779 (2)	169 (2)
O2*W*—H4*W*···O1^v^	0.82 (2)	2.06 (2)	2.869 (2)	168 (2)

Symmetry codes: (i) −*x*+1, −*y*+1, −*z*+1; (ii) −*x*+1/2, *y*+1/2, −*z*+3/2; (iii) −*x*+1, −*y*+2, −*z*+1; (iv) *x*+1/2, −*y*+1/2, *z*+1/2; (v) −*x*+1/2, *y*−1/2, −*z*+1/2.
